# Abnormal Curtain Signs Identified With a Novel Lung Ultrasound Protocol in Six Dogs With Pneumothorax

**DOI:** 10.3389/fvets.2019.00291

**Published:** 2019-08-28

**Authors:** Søren Boysen, Jantina McMurray, Kris Gommeren

**Affiliations:** ^1^Department of Veterinary Clinical and Diagnostic Sciences, Faculty of Veterinary Medicine, University of Calgary, Calgary, AB, Canada; ^2^Faculty of Veterinary Medicine, University of Liège, Liège, Belgium

**Keywords:** curtain sign, point of care ultrasound, pneumothorax, FAST, lung ultrasound

## Abstract

Pneumothorax is typically ruled out sonographically by detecting a glide sign, lung pulse, and/or B lines, and ruled in by detecting the return of a glide sign and/or presence of a lung point. This case series describes novel lung ultrasound findings (abnormal curtain signs) in dogs with naturally-occurring pneumothorax. This case series also describes a novel lung ultrasound protocol that involves evaluating the curtain sign along the entire thoracoabdominal border and evaluating the ventral pleural space with the probe parallel to the ribs. Six dogs with pneumothorax (three traumatic pneumothorax and three spontaneous pneumothorax) had lung ultrasound performed. All dogs had normal synchronous curtain signs in the caudal mid-to-ventral region of the thorax and abnormal curtain signs in the caudal mid-to-dorsal thoracic regions. Five dogs had bilateral pneumothorax; four had a lung point and absence of a glide sign bilaterally, and one had a lung point identified unilaterally (a lung point was not visible on the opposite side and the glide sign was equivocal bilaterally). One dog had a unilateral pneumothorax, in which a lung point and absence of a glide sign were identified. With the probe parallel to the ribs in the ventral thorax, a small volume pleural effusion was also identified in two dogs. All dogs had mild to moderate quantities of pleural air removed via thoracentesis or chest tubes following lung ultrasound. Two distinct types of abnormal curtain sign were observed, referred to as the asynchronous curtain sign and the double curtain sign. The authors hypothesize that these abnormal curtain signs are caused by the presence of free air within and/or cranial to the costophrenic recess. To the authors' knowledge, this is the first description of pneumothorax-induced abnormal curtain signs, and the first report of evaluating the curtain sign to diagnose pneumothorax in any species. Further research is required to determine the sensitivity and specificity of asynchronous and double curtain signs in diagnosing pneumothorax, and to investigate whether probe orientation parallel to the ribs in the ventral thorax will improve detection of pleural effusion.

## Introduction

In veterinary medicine, the accuracy of diagnosing pneumothorax with lung ultrasound is variable, and likely depends on the experience of the sonographer, underlying pathology, patient positioning, scanning protocol, and criteria used to diagnose pneumothorax (1–5).

Historically, pneumothorax is sonographically ruled out by detecting a glide sign, lung pulse (rhythmic movement of the pleura synchronous with the heart beat), and/or B lines, and ruled in by detecting the return of a glide sign and/or presence of a lung point (4, 5).

Sonographically, the curtain sign is a sharply demarcated vertical edge separating aerated lung from abdominal contents. Lung ultrasound protocols generally avoid evaluation of the curtain sign because the thoracoabdominal border is reportedly difficult to interpret and abdominal structures may be confused for lung pathology (5–7). However, the human literature has recently described abnormal curtain signs associated with a number of pleural space and lung pathologies (8–10). When pleural effusion or lung consolidation occupies the space between the parietal lining and the diaphragm, the normal aerated lung/soft tissue interface (curtain sign) is lost (8, 9). With diaphragmatic hernias, a double curtain sign can occur when herniated abdominal contents located within the pleural space are trapped between aerated lung and the diaphragm (10); a cranial curtain sign is observed where aerated lung and herniated abdominal content are in contact, and a second, caudal curtain sign, occurs where herniated abdominal content contacts the diaphragm. Finally, massive pneumothorax can create a pseudo curtain sign, which has the appearance of a normal curtain sign (8); the pseudo curtain sign is believed to occur when large or complete pneumothoraxes result in sufficient free air in the costophrenic recess to create a similar vertical air/soft tissue interface to that of aerated lung and soft tissue. Pseudo curtain signs appear to move primarily due to diaphragmatic excursions that allow air to move in and out of the costophrenic recess. The curtain sign has not been assessed in small animals.

The objectives of the current case series are: (1) describe a novel lung ultrasound protocol; (2) describe the finding of two distinct types of abnormal curtain sign in dogs with naturally occurring pneumothorax; and (3) propose a potential hypothesis for why abnormal curtain signs occur with pneumothorax. To the authors' knowledge, this is the first report of a pneumothorax-induced abnormal curtain sign in any species.

## Lung Ultrasound Protocol

**Figure 1:**
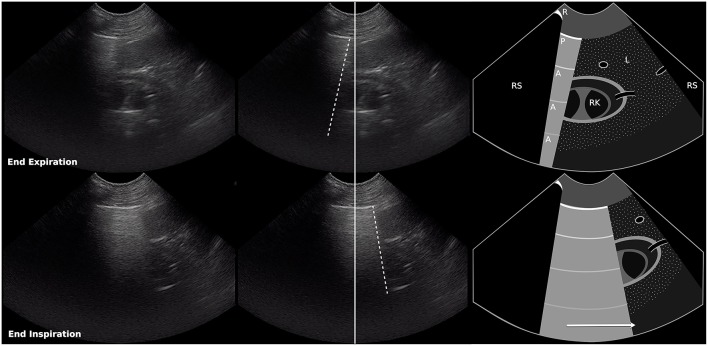
Still images and schematic of a normal curtain sign at end expiration (top row) and end inspiration (bottom row). The ultrasound probe was positioned over the right paralumbar region at the level of the right kidney, over the liver, between the 11th and 12th intercostal spaces, and incorporates the air/soft tissue interface between the abdomen and the thorax which appears as a vertical edge (curtain sign). During inspiration, the vertical edge of the curtain sign and the abdominal contents move caudally at a similar rate. Dashed lines, vertical edge (air/soft tissue interface); Solid line, center of image; R, rib; RS, rib shadow; P, pleural line; A, A line; L, liver; RK, right kidney. Arrow shows direction of movement of the vertical edge during inspiration (also see Video S1).

**Figure 2:**
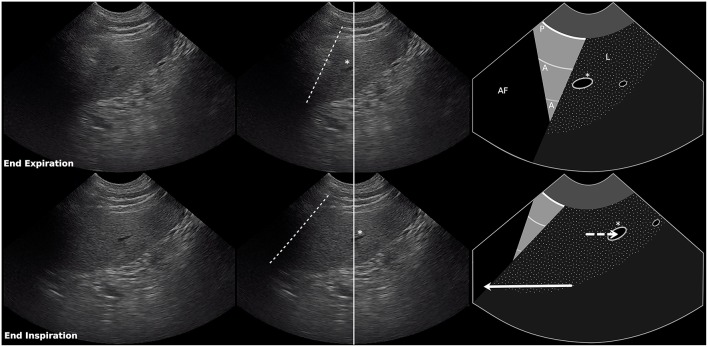
Still images and schematic of an asynchronous curtain sign at end expiration (top row) and end inspiration (bottom row). The ultrasound probe was positioned over the right paralumbar region just below the right kidney, over the liver, between the 9th and 10th intercostal spaces, and incorporates the air/soft tissue interface between the abdomen and the thorax which appears as a vertical edge (curtain sign). During inspiration, the vertical edge of the curtain sign moves in the opposite direction (cranially) of the abdominal contents (caudally). Dashed lines, vertical edge (air/soft tissue interface); Solid line, center of image; RS, rib shadow; P, pleural line; A, A line; L, liver; Asterisk, hepatic vessel. Solid arrow shows direction of movement of the vertical edge during inspiration. Dashed arrow shows direction of movement of the abdominal contents during inspiration. Length of arrow represents degree of movement relative to the image above (also see Video S2).

**Figure 3:**
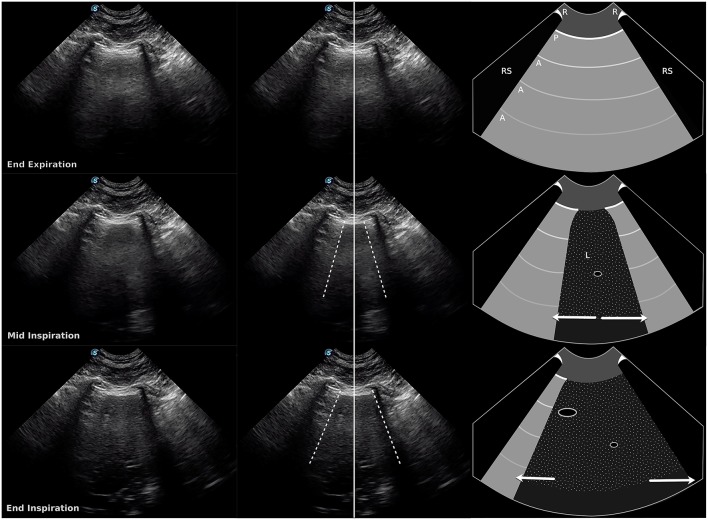
Still images and schematic of a double curtain sign at end expiration (top row), mid-inspiration (middle row), and end inspiration (bottom row). The ultrasound probe is located over the liver, about halfway up the thorax, between the 7th and 8th intercostal spaces, over the air/soft tissue interface between the abdomen and the thorax. During inspiration, two vertical edges of the curtain sign are visible in the same sonographic window. Dashed lines, vertical edges (air/soft tissue interface); Solid line, center of image; R, rib; RS, rib shadow; P, pleural line; A, A line; L, liver. Arrows show movement of the vertical edges in opposite directions during inspiration in this example. Length of arrow represents degree of movement relative to the image above (also see Video S3).

In healthy patients, the curtain sign is the sharply demarcated vertical edge separating aerated lung from abdominal contents (Figure 1). During inspiration, the vertical edge and abdominal contents move caudally at a similar rate; the movement of the vertical edge and abdominal contents is therefore synchronous (Video S1) (7–9). With pneumothorax, an abnormal curtain sign is the vertical edge separating free air in the caudal pleural space from abdominal contents. We define the asynchronous curtain sign as movement of the vertical edge in the opposite direction of the abdominal contents or minimal movement of the vertical edge while abdominal contents move caudally (Figure 2 and Video S2). We define the double curtain sign as two vertical edges visible in the same sonographic window (Figure 3 and Video S3).

**Figure 4:**
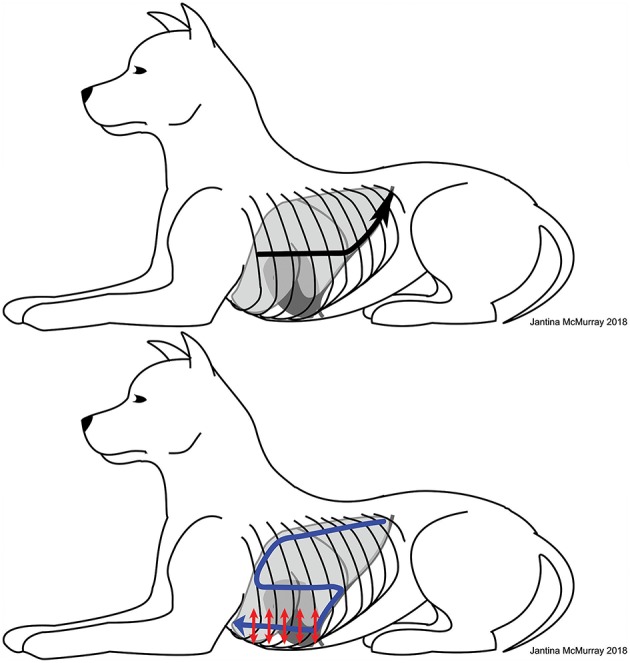
Illustration of the modified Armenise lung ultrasound protocol. Upper image: the ultrasound probe is first placed perpendicular to the ribs, ~1/2 to 2/3 of the way up the thorax, just caudal to the scapula in the 6th intercostal space (widest part of the thorax). The black arrow indicates initial probe positioning followed by sliding of the probe caudally (until the curtain sign is identified), followed by sliding of the probe dorsally along the curtain sign until the hypaxial muscles are identified (loss of the pleural line). The probe is then slowly slid ventrally off the hypaxial muscles until the pleural line reappears (the most caudal dorsal region of the thorax). Lower image: after identifying the most caudal dorsal location, the remainder of the lung and pleural space is evaluated in a similar fashion as described by Armenise et al. (3) and follows an S-shaped pattern (blue arrow), with the exception that the curtain sign is identified again in the mid thorax and followed ventrally until it contacts the heart, at which point the probe is turned parallel to the ribs to evaluate the most ventral regions of the thorax for the presence of pleural effusion. The probe can be slid both ventrally and dorsally within each intercostal space with the probe remaining parallel to the ribs to look for lung pathology as well as pleural effusions in the ventral region of the pleural space (red arrows indicate parallel probe positioning with small ventral and dorsal sliding of the probe).

A modified Armenise lung ultrasound protocol (3) was developed to evaluate the curtain sign at the caudal borders of the lung for pathology, to evaluate regions of the thorax where free air is most likely to accumulate (caudal dorsal thorax with the patient in sternal recumbency or standing, widest part of the thorax at the caudal border with patients in lateral recumbency), and to evaluate the ventral-most regions of the thorax for small volumes of pleural effusion that may not be identified when the probe remains perpendicular to the ribs (Figure 4). Dogs were scanned in sternal, right lateral, and left lateral recumbency as well as standing. Fur was not clipped and alcohol was used as the coupling agent. The ultrasound probe is first placed perpendicular to the ribs, ~1/2 to 2/3 of the way up the thorax, just caudal to the scapula in the 6th intercostal space (widest part of the thorax). The probe is then slid horizontally in a caudal direction one intercostal space at a time until the curtain sign is identified. If the curtain sign is normal at this site, the probe is slid dorsally (keeping the curtain sign visible within the sonographic window) until the hypaxial muscles are encountered (as indicated by loss of the pleural line). The probe is then slid ventrally until the pleural line reappears (the most caudal dorsal region of the thorax). From this point, the remainder of the lung and pleural space is evaluated in a similar fashion as described by Armenise et al. (3), with the exception that the curtain sign is identified again in the mid thorax and followed ventrally until it contacts the heart, at which point the probe is turned parallel to the ribs to evaluate the most ventral regions of the thorax for the presence of pleural effusion (Figure 4).

Ethics approval for lung ultrasound and thoracic radiographs was obtained from the University of Calgary as part of a larger study, and owner consent was obtained for inclusion of all six dogs.

## Case 1

A 4-year-old male castrated Samoyed (13 kg) was referred with a radiographic diagnosis of spontaneous pneumothorax. On presentation the dog was moderately dyspneic. Pneumothorax was confirmed with three-view thoracic radiographs, thoracentesis was performed, and bilateral small-bore chest tubes (MILA thoracostomy tube, MILA International, Inc., Erlanger, KY.) were placed with manual suctioning every 4 h. Twenty-four hours after arrival, computed tomography (CT) confirmed the diagnosis of bilateral pneumothorax. The dog underwent a median sternotomy and a partial left lung lobectomy. Post-surgical chest tubes were maintained. Eight hours after surgery, the dog was tachypneic (72 bpm) and mildly dyspneic. Lung ultrasound, performed in a standing position, identified a normal synchronous curtain sign ventrally on both sides of the thorax and both an asynchronous curtain sign and a double curtain sign in the mid-to-dorsal caudal lung regions bilaterally. A glide sign was absent dorsally and a lung point was identified bilaterally. Following lung ultrasound, 11 and 9 mL/kg of air was removed from each chest tube. The dog was eventually discharged.

## Case 2

A 5-year-old female spayed Collie cross (25 kg) was referred with a radiographic diagnosis of spontaneous pneumothorax. On presentation the dog was tachypneic (56 bpm) and mildly dyspneic. Bilateral pneumothorax was confirmed with three-view thoracic radiographs and CT. The right accessory lung lobe and caudal portion of left cranial lung lobe were removed during exploratory median sternotomy. Bilateral post-surgical small-bore chest tubes were placed. The day after surgery, the dog was tachypneic (80 bpm) and mildly dyspneic. Lung ultrasound, performed in sternal recumbency, identified bilateral normal synchronous curtain signs ventrally and an asynchronous curtain sign and a double curtain sign in the mid-to-dorsal caudal lung regions bilaterally. A glide sign was absent dorsally and a lung point was identified bilaterally. Following lung ultrasound, 8 and 18 mL/kg of air was removed from each chest tube. The dog was eventually discharged.

## Case 3

An 8-year-old female spayed Husky cross (18 kg) was referred with a radiographic diagnosis of spontaneous pneumothorax. On presentation, the dog was eupnic (28 bpm). Lung ultrasound, performed in a standing position, identified bilateral normal synchronous curtain signs ventrally and an asynchronous curtain sign and a double curtain sign in the mid-to-dorsal caudal lung regions bilaterally. A glide sign was absent dorsally and a lung point was identified bilaterally. Immediately following lung ultrasound, three-view thoracic radiographs confirmed a mild to moderate pneumothorax. Sixty hours after arrival, a CT revealed moderate bilateral pneumothorax. A median sternotomy was performed, and a single bulla was removed via partial left lung lobectomy. The dog was eventually discharged.

## Case 4

A 10-month-old male mixed breed dog (38 kg) was referred with pelvic fractures and a radiographic diagnosis of pneumothorax following motor vehicle trauma. On presentation the dog was tachypneic (100 bpm) and moderately dyspneic. Eighteen milliliters per kilogram of air was removed from the left hemithorax with thoracentesis. Eight hours later, the dog was mildly tachypneic and dyspneic. Lung ultrasound, performed in sternal recumbency, identified bilateral normal synchronous curtain signs ventrally and an asynchronous curtain sign and a double curtain sign in the mid-to-dorsal caudal lung regions bilaterally. The glide sign was difficult to assess bilaterally and considered equivocal. On the right hemithorax, a lung point was present in the mid caudal region and there were increased B lines at and below the lung point. On the left hemithorax, a lung point and B lines were not observed. With the probe oriented parallel to the ribs, a scant amount of pleural effusion was noted bilaterally in the most ventral regions of the pleural space abutting the diaphragm. Following lung ultrasound, three-view thoracic radiographs revealed a mild pneumomediastinum, mild pneumothorax, and a small volume of pleural effusion. Concurrent injuries included right caudal acetabular fracture and multiple sacral fractures. The dog was eventually discharged.

## Case 5

An 18-month-old female spayed Great Pyrenees (38 kg) was referred with right humeral fracture and a radiographic diagnosis of pneumothorax following motor vehicle trauma. On presentation the dog was tachypneic (84 bpm) and moderately dyspneic. Thoracentesis was performed (54 mL/kg of air removed from right hemithorax and 40 mL/kg from left hemithorax). A right sided small-bore chest tube with continuous suction was placed. Twelve hours post-presentation, continuous suction was discontinued and lung ultrasound was performed in left lateral recumbency to assess the right hemithorax and partial sternal recumbency to assess the left hemithorax (the humeral fracture prevented sternal positioning due to patient discomfort). A bilateral normal synchronous curtain sign was observed ventrally and an asynchronous curtain sign and a double curtain sign were present in the mid-to-dorsal caudal lung regions bilaterally. A glide sign was absent dorsally and a lung point was identified bilaterally. Increased numbers of B lines were observed in the ventral lung regions at and below the lung point bilaterally. With the probe oriented parallel to the ribs, a scant amount of pleural effusion was noted bilaterally in the most ventral regions of the pleural space. Following lung ultrasound, 8 mL/kg of air was removed through the chest tube and three-view thoracic radiographs showed mild pneumothorax bilaterally, a small volume of pleural effusion, and increased soft tissue opacity bilaterally consistent with pulmonary contusions. A left brachial plexus avulsion was also diagnosed 12 h following admission. The owner elected euthanasia.

## Case 6

A 2-year-old male neutered mixed breed dog (18 kg) was evaluated following motor vehicle trauma. The dog was tachypneic (68 bpm) and moderately dyspneic. Lung ultrasound, performed in sternal recumbency, identified bilateral normal synchronous curtain signs ventrally. On the left hemithorax, both an asynchronous curtain sign and a double curtain sign were present in the mid-to-dorsal caudal lung regions, the glide sign was absent dorsally, a lung point was identified, and increased numbers of B lines were observed in the ventral lung regions at and below the lung point. On the right hemithorax, a normal synchronous curtain sign was present along the entire caudal border of the thorax, a glide sign was present at the most caudal dorsal regions, and diffuse B lines were observed in the mid to ventral lung regions. Following lung ultrasound, thoracentesis was performed only on the left hemithorax (28 mL/kg of air removed). Following thoracentesis, three-view thoracic radiographs confirmed a moderate left-sided pneumothorax, multiple left-sided rib fractures, and increased soft tissue opacity bilaterally consistent with pulmonary contusions. Concurrent injuries included a degloving tail injury and a left coxofemoral luxation. The dog was eventually discharged.

## Discussion

Abnormal curtain signs were identified in six dogs presenting with pneumothorax using a novel point-of-care lung ultrasound protocol developed to assess the thoracoabdominal border. All dogs had two distinctly different abnormal curtain signs: the asynchronous curtain sign and the double curtain sign. In all dogs, abnormal curtain signs were only observed in the mid-to-dorsal caudal regions of the thorax. Normal synchronous curtain signs were present in the mid-to-ventral caudal regions of the same hemithorax in all dogs.

**Figure 5:**
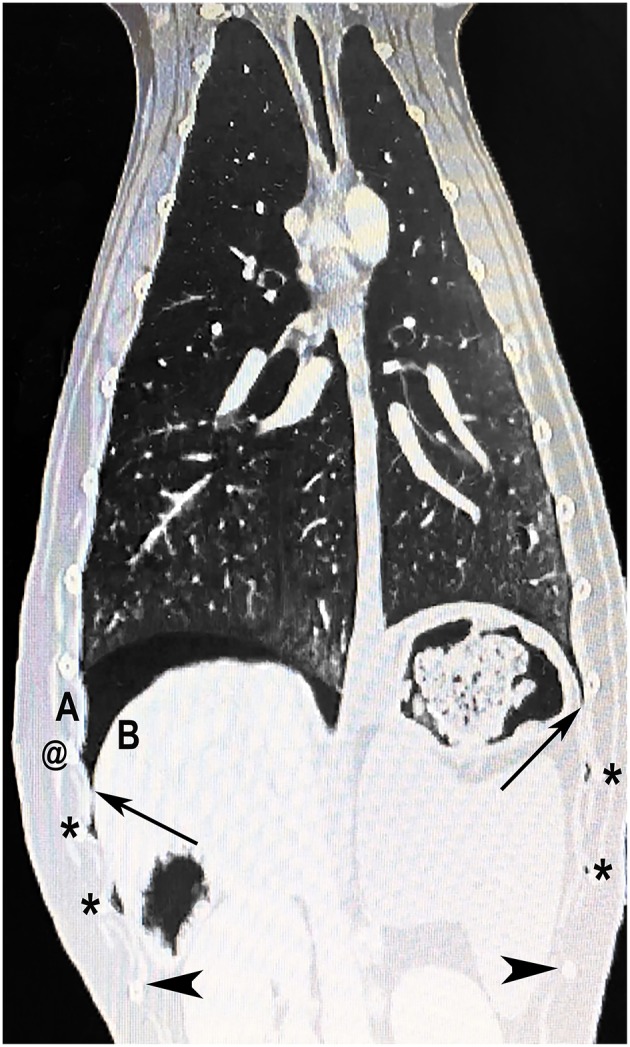
Hypothesized mechanism of asynchronous and double curtain signs. Computed tomography image of a dog with spontaneous pneumothorax showing free air (asterisks) trapped in the costophrenic recess. Cranial and caudal borders of the costophrenic recess are indicated by long arrows and arrowheads, respectively. An asynchronous curtain sign occurs when there is free air cranial to the costophrenic recess (cranial to long black arrows); during inspiration, the negative pressure created by expansion of the thorax pulls the costal diaphragm (B) laterally until it contacts the ribcage (A) and pushes free air cranially, while contraction of the diaphragm pushes abdominal contents caudally. A double curtain sign occurs when the ultrasound probe is placed between two pockets of air (between two asterisks) creating two visible vertical edges (air/soft tissue interfaces). A double curtain sign can also appear as a result of air becoming trapped in the costrophrenic recess during inspiration. For example, if negative pleural pressure pulls the costal diaphragm (B) into contact with the thoracic wall (A), air may become trapped at the @ symbol, which would create a double curtain sign when the probe is placed between the cranial asterisk and the @ symbol. The leading edge of the double curtain sign in this situation may also be asynchronous, moving cranially during inspiration as the diaphragm continues to come in contact with the chest wall.

The diaphragm is dome-shaped and bulges cranially into the thoracic cavity, so the costal aspect of the diaphragm is pushed directly against the inner surface of the last few ribs, forming the costophrenic recess (11, 12). During inspiration the lung expands caudally into the costophrenic recess and contraction of the diaphragm displaces abdominal contents caudally (13), creating a curtain sign with a single vertical edge that moves caudally at a similar rate as the abdominal structures (Figure 1 and Video S1). We hypothesize that abnormal curtain signs occur with pneumothorax due to the presence of free air within and/or cranial to the costophrenic recess (Figure 5). An asynchronous curtain sign may result when there is free air at the cranial border of the costophrenic recess. During inspiration, the negative pressure created by expansion of the thorax may pull the costal diaphragm laterally until it contacts the ribcage and pushes free air cranially, while contraction of the diaphragm pushes abdominal contents caudally. This creates asynchrony between the vertical edge moving cranially and abdominal contents moving caudally (Figure 2 and Video S2). It is also possible that during inspiration, the negative pleural pressure is insufficient to pull the costal diaphragm into contact with the ribcage. In this situation, minimal movement of free air combined with caudal movement of abdominal contents would create an asynchronous curtain sign where the vertical edge remains relatively motionless. A double curtain sign may result when free air is trapped within the costophrenic recess. This free air trapping creates an area where the diaphragm is in contact with the chest wall and air is present both cranial to that area (either aerated lung, free air in the pleural space, or an air bubble trapped in the costophrenic recess cranial to the area of contact) and caudal to that area (air bubble trapped in the costophrenic recess caudal to the area of contact). This would create a double curtain sign with two vertical edges separating air from abdominal contents (Figures 3, 5 and Video S3).

Although a pseudo curtain sign has been described in humans with pneumothorax, a pseudo curtain was not identified in any dogs of the current study. This is likely due to the fact that all dogs in the current series had mild to moderate pneumothoraxes. It is possible that with more severe or complete pneumothoraxes, a pseudo curtain sign will be seen in dogs as it is in humans.

The asynchronous curtain sign is likely a similar phenomenon to reverse sliding recently reported in anesthetized dogs with experimentally-induced pneumothorax (4). Reverse sliding refers to the direction of movement of A lines. At the thoraco-abdominal border of healthy dogs, A lines slide caudally on inspiration as the diaphragm moves caudally, and cranially on expiration as the diaphragm moves cranially (4). With mild pneumothorax, A lines move in the opposite direction, sliding cranially on inspiration and caudally on expiration (4). Reverse sliding was found to be more sensitive and specific than other lung ultrasound findings (M mode barcode or stratosphere sign, lung slide, lung point, and lung pulse) for the diagnosis of mild pneumothorax (4). Dogs in the current case series were not evaluated for reverse sliding. However, given the similarities in the description of reverse sliding and the asynchronous curtain sign, we suspect that the presence of an asynchronous curtain sign in which the vertical edge moves cranially during inspiration would be associated with the presence of reverse sliding. An asynchronous curtain sign in which the vertical edge is relatively motionless would not be expected to result in reverse sliding. The double curtain sign may or may not be associated with reverse sliding. Therefore, abnormal curtain signs may still be present when reverse sliding is not detectable. Furthermore, given that both abnormal curtain signs were identified in all six dogs while the absence of a glide sign was equivocal in two instances and the lung point was not identified in one instance, abnormal curtain signs may be more sensitive than historical lung ultrasound findings used to diagnose mild to moderate pneumothorax. Further research is required to investigate the sensitivity and specificity of reverse sliding as well as abnormal curtain signs, and to investigate whether the volume of air in the pleural space affects the location of abnormal curtain signs along the caudal thoracoabdominal border.

The sensitivity of T-FAST is reported to be moderate for the detection of pleural fluid when compared to CT in dogs suffering trauma (1). Most thoracic point-of-care ultrasound protocols in small animals recommend orienting the probe perpendicular to the ribs (3, 5, 6). In two dogs that suffered from trauma in the current case series, small volume pleural effusion in the most ventral regions of the pleural space was identified only with the probe orientated parallel to the ribs. Both of these cases had scant pleural effusion also identified on thoracic radiographs. Further research is required to investigate if probe orientation (perpendicular vs. parallel to the ribs) in the ventral regions of the thorax will improve the detection of pleural effusion with veterinary point-of-care ultrasound.

There are several limitations to this case series. Only a small number of spontaneously breathing cases (*n* = 6) were included, and only mild to moderate cases of pneumothorax were assessed. The sensitivity and specificity of abnormal curtain signs for diagnosing pneumothorax was not evaluated. The clinician performing the lung ultrasound protocols was not blinded to possible underlying pathology and could have been biased in searching for sonographic findings of pneumothorax. However, each patient was thoroughly evaluated for the absence of a glide sign, lung point, return of a glide sign, and presence of abnormal curtain signs.

This case series describes a novel lung ultrasound protocol that involves scanning the entire thoracoabdominal border and evaluating the curtain sign. This case series also describes the novel finding of abnormal curtain signs in dogs with naturally occurring pneumothorax. Abnormal curtain signs included the asynchronous curtain sign and the double curtain sign. We hypothesize that these abnormal curtain signs are caused by the presence of free air within and/or cranial to the costophrenic recess.

## Data Availability

All datasets generated for this study are included in the manuscript/Supplementary Files.

## Author Contributions

SB scanned all patients, saved all cineloops, collected all data, wrote first draft of the manuscript, produced CT image, wrote figure legend for CT image. JM reviewed all cineloops, heavily revised/rewrote first draft and added significant concepts to the discussion, created Figures 1–3 and wrote figure legends for Figures 1–3. KG reviewed cineloops and critically reviewed second draft of the paper.

### Conflict of Interest Statement

The authors declare that the research was conducted in the absence of any commercial or financial relationships that could be construed as a potential conflict of interest.

## Supplementary Material

Video S1 Video depicting a normal curtain sign.

Video S2 Video depicting an asynchronous curtain sign.

Video S3 Video depicting a double curtain sign.

## References

[1] Walters AM O'Brien MA Selmic LE Hartman S McMichael M O'Brien RT. Evaluation of the agreement between focused assessment with sonography for trauma (AFAST/TFAST) and computed tomography in dogs and cats with recent trauma. J Vet Emerg Crit Care. (2018) 28:429–35. 10.1111/vec.12732 29901282

[2] Cole L Pivetta M Humm K Evaluation of the Agreement between a Lung Ultrasound Protocol, Vet Blue, and Thoracic Computed Tomography in Dogs and Cats. Tallinn: Journal of Veterinary Emergency and Critical Care (2019).

[3] Armenise A Boysen S Rudloff E Neri L Spattini G Storti E. Veterinary-focused assessment with sonography for trauma-airway, breathing, circulation, disability and exposure: a prospective observational study in 64 canine trauma patients. J Small Anim Pract. (2019) 60:173–82. 10.1111/jsap.12968 30549049

[4] Hwang TS Yoon YM Jung DI Yeon SC Lee HC. Usefulness of transthoracic lung ultrasound for the diagnosis of mild pneumothorax. J Vet Sci. (2018) 19:660–6. 10.4142/jvs.2018.19.5.660 30041286 PMC6167337

[5] Lisciandro GR Lagutchik MS Mann KA Voges AK Fosgate GT Evaluation of a thoracic focused assessment with sonography for trauma (TFAST) protocol to detect pneumothorax and concurrent thoracic injury in 145 traumatized dogs. J Vet Emerg Crit Care. (2008) 18:258 10.1111/j.1476-4431.2008.00312.x

[6] Lisciandro GR1 Fosgate GT Fulton RM. Frequency and number of ultrasound lung rockets (B-lines) using a regionally based lung ultrasound examination named vet BLUE (veterinary bedside lung ultrasound exam) in dogs with radiographically normal lung findings. Vet Radiol Ultrasound. (2014) 55:315–22. 10.1111/vru.12122 24382172

[7] Boysen SR AFAST and TFAST in the intensive care unit. In: Silverstein DC Hopper K editors. Small Animal Critical Care Medicine. 2nd ed. St. Louis: Saunders (2015). p. 993. 10.1016/B978-1-4557-0306-7.00189-6

[8] Lee, FCY The curtain sign in lung ultrasound. J Med Ultrasound. (2017) 25:101–4. 10.1016/j.jmu.2017.04.005 30065468 PMC6029316

[9] Lee, FCY Lung ultrasound—a primary survey of the acutely dyspneic patient. J Int Care. (2016) 4:57 10.1186/s40560-016-0180-1 PMC5007698 27588206

[10] Conte EG Gerardi RE Smargiassi A Gatto A Valentini P Nanni L. A 3-year-old child with a history of persistent dry cough and fever. Chest. (2017) 151:e127–9. 10.1016/j.chest.2016.12.036 28599945

[11] Slatter D Functional anatomy of the respiratory system. In: Slatter D editor. Textbook of Small Animal Surgery: Volume 1. 3rd ed. Philadelphia, PA: Saunders (2003). p. 778.

[12] Evans HE de Lahunta A The muscular system: muscles of the thoracic wall (Musculi Thoracis). In: Evans HE de Lahunta A editors. Miller's Anatomy of the Dog. 4th ed. St. Louis, MO: Saunders (2013). p. 223.

[13] Dyce KM Sack WO Wensing CJG The locomotor apparatus and the respiratory apparatus. In: Dyce KM editor. Textbook of Veterinary Anatomy. 4th ed. St. Louis, MO: Saunders (2010). p. 159.

